# Single-shot ptychography at a soft X-ray free-electron laser

**DOI:** 10.1038/s41598-022-18605-x

**Published:** 2022-08-24

**Authors:** Konstantin Kharitonov, Masoud Mehrjoo, Mabel Ruiz-Lopez, Barbara Keitel, Svea Kreis, Seung-gi Gang, Rui Pan, Alessandro Marras, Jonathan Correa, Cornelia B. Wunderer, Elke Plönjes

**Affiliations:** 1 Deutsches Elektronen-Synchrotron DESY, Hamburg, Germany; 2 Center for Free-Electron Laser Science CFEL, Deutsches Elektronen-Synchrotron DESY, Hamburg, Germany

**Keywords:** Optics and photonics, Optical physics, Techniques and instrumentation

## Abstract

In this work, single-shot ptychography was adapted to the XUV range and, as a proof of concept, performed at the free-electron laser FLASH at DESY to obtain a high-resolution reconstruction of a test sample. Ptychography is a coherent diffraction imaging technique capable of imaging extended samples with diffraction-limited resolution. However, its scanning nature makes ptychography time-consuming and also prevents its application for mapping of dynamical processes. Single-shot ptychography can be realized by collecting the diffraction patterns of multiple overlapping beams in one shot and, in recent years, several concepts based on two con-focal lenses were employed in the visible regime. Unfortunately, this approach cannot be extended straightforwardly to X-ray wavelengths due to the use of refractive optics. Here, a novel single-shot ptychography setup utilizes a combination of X-ray focusing optics with a two-dimensional beam-splitting diffraction grating. It facilitates single-shot imaging of extended samples at X-ray wavelengths.

## Introduction

X-ray ptychography is a scanning coherent diffraction imaging (CDI) method capable of high-resolution imaging of extended objects^1^. Ptychography is based on measuring multiple diffraction patterns while scanning a photon beam over a sample. The acquired patterns are evaluated in a phase retrieval procedure to reconstruct the complex beam wavefield (hereafter called probe) and, at the same time, the object transmission function. Scanning is performed such that it achieves a controlled degree of overlap between adjacent scan points. A high degree of overlap increases the convergence stability of the reconstruction, prevents reconstruction ambiguities, and results in redundancy in the measured data enabling simultaneous reconstruction of both sample and probe.

Recently, classical (scanning-based) ptychography was successfully implemented at X-ray free-electron laser (FEL) light sources for both imaging applications and photon beam characterization^2–4^. The ultra-high brightness and the spatial coherence of the FEL radiation significantly decrease the data acquisition time for both, full-field and scanning imaging techniques. The femtosecond length of the FEL pulses can enable imaging of dynamical processes with unprecedented temporal resolution. However, ptychography in its conventional scanning mode cannot capture the dynamics of systems. Implementation of single-shot ptychography using X-rays, on the other hand, can greatly enhance the potential of imaging at FEL sources and enable diffraction-limited time-resolved imaging of extended samples and dynamics of complex matter in the X-ray regime.

Single-shot ptychography was first proposed by Sidorenko et al.^5^ and was further expanded to Fourier^6^, multi-slice^7^ and multi-wavelength^8^ regimes in the visible wavelength range. A setup based on a 4-f lens arrangement is capable of performing simultaneous illumination of a sample by multiple beamlets in one shot. It utilizes a pinhole array placed in the front focal plane of the first lens as a light source. The sample is placed close to the common focal plane of the two lenses. The overlap of the beamlets produced by the individual pinholes can be adapted by adjusting the focus-sample distance. However, this concept cannot be directly transferred to the extreme ultraviolet (XUV) and X-ray regimes due to the lack of efficient high numerical aperture refractive optics at these wavelengths.

**Figure 1:**
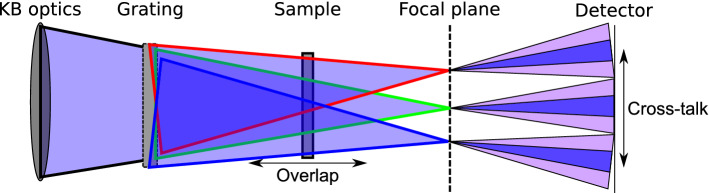
A schematic 2D diagram of the proposed experimental setup. The FEL beam is focused downstream of the sample by the pair of KB mirrors. The beam-splitting grating is placed in front of the sample and splits the beam into several diffraction orders (shown in red, green, and blue). The sample, placed in the vicinity of the grating, is illuminated by the overlapping beamlets produced by the grating. The degree of overlap and the field of view in the sample plane can be controlled by changing the grating-sample distance. The detector is placed downstream of the focal plane. The focal length of the KB mirrors is selected such that a separation of the beamlets is facilitated (dark blue) and possible cross-talk between the radiation scattered by the sample minimized (light-purple).

Alternatively, we propose a setup based on combining bendable Kirkpatrick–Baez (KB) optics for the XUV and soft X-ray range with a beam-splitting two-dimensional (2D) diffraction grating inspired by the work of Pan et al.^9^. The schematic diagram of the proposed setup is shown in Fig. 1. This setup allows illuminating the sample by multiple beamlets in one shot. The test object is placed in the vicinity of the beam-splitting diffraction grating and the grating splits the incoming FEL beam into a number of beamlets (shown in red, green, and blue in Fig. 1). The degree of overlap of the beamlets and the field of view (FOV) (i.e. the total area of illumination on the sample) is determined by the specific design of the grating. Moreover, it is possible to optimize both: the degree of overlap of the beamlets on the sample and the FOV. The former is adjusted by changing the grating-sample distance, while the latter is adapted by varying the focal length of the bendable KB mirrors^10,11^.

For the image reconstruction we used an automatic differentiation^12^ (AD) powered ptychography engine described in our previous work^2^. AD is capable of numerically obtaining the gradients of any differentiable function with respect to its arguments. This allows splitting the ptychography reconstruction into three independent parts: first, a forward model describing the intensity formation at the detector plane and allowing the approximation of the measured intensity, second, a loss function qualitatively evaluating the difference between the measured and approximated intensities, and finally, a gradient-based optimizer which utilizes the gradients of the loss function to minimize it and find the appropriate values for sample, probe, and other reconstructable parameters. As an experimental proof of concept, the single-shot ptychography technique was demonstrated at the FLASH2^13^ variable micro-focus beamline FL24^10^.

## Methods

### Ptychography formalism

In classical scanning ptychography, a sample *O* is scanned by a probe *P* in numerous positions characterized by the displacement vectors $\mathbf{r} _j, j\in 1\ldots N$ where *N* is the number of scan points. Under the thin sample approximation^14^, the exit-wave $\Psi _j$ produced at the $j-$th position may be expressed as $\Psi _j = P \cdot O_\mathbf{r _{j}}$. The intensity produced by the exit-wave at the detector may be calculated as:

1 $$\begin{aligned} I_j = \left| \mathscr {P}\{\Psi _j \} \right| ^2, \end{aligned}$$

where $\mathscr {P}$ is the propagator defined by the geometry of the experiment and the Fresnel number^15^. The Fresnel number is given as $\frac{a^2}{\lambda z}$, where *a* is the characteristic size of the illuminated area, *z* is the propagation distance and $\lambda$ is the wavelength of the radiation. During the ptychography reconstruction, multiple intensity patterns are used to perform phasing and to reconstruct the complex-valued *P* and *O* functions.

This task can be viewed as an optimization task of finding a pair of *P* and *O* that minimizes a loss function $\mathscr {E}(I_j,\tilde{I}_j)$ evaluating the similarity between $I_j$ (measured) and $\tilde{I}_j$ (estimated) intensity at the *j*-th scan position. The minimization can be performed by gradient-based optimization methods^16^ utilizing automatic differentiation. AD is capable of dynamically estimating gradients of any differentiable function with respect to its parameters. This makes a ptychographic reconstruction feasible when an appropriate differentiable forward model describing the particular experiment is provided^2,17^. The additional important components of the AD-powered ptychography reconstruction are a loss function and a gradient-based optimizer. A loss function evaluates the quality of reconstruction by comparing the approximated and measured intensity distributions. Additionally, various regularization terms can be included in the loss function to perform the denoising and speed up the convergence. In this work, the loss function is used in the following form:

2 $$\begin{aligned} L(I,\tilde{I},O) = \mathscr {E}(I,I) + TVD(O) = \frac{1}{N_d}\sum ^{N_d}_{m} \left\Vert {\sqrt{I_m} -\sqrt{\tilde{I}_m} }\right\Vert ^2 + \gamma \sum _{m}^{N_d}{\sqrt{\left| \nabla _x O \right| ^2_m + \left| \nabla _y O \right| ^2_m} } \end{aligned}$$

where $\tilde{I}$ is the approximation of the intensity provided by the forward model, *I* is the measured intensity, $N_d$ is the total number of pixels of the used 2D detector, *N* is the number of pixels in a specific row or column, $\gamma$ is the regularization weight which should be selected individually to ensure the stability of the reconstruction, $\nabla _x O,\nabla _y O$ represent horizontal and vertical gradients of the object image, respectively, and *TVD* stands for the total variation denoising regularizer^18^.

Although initially ptychography was developed under the assumption of a fully-coherent and spatially stable beam, the actual X-ray probe beam may not fully meet these requirements and necessitate a modification of the forward model. Potentially, an only partial spatial coherence of the probe must be taken into account as proposed by Thibault et al.^19^. In this case, the probe can be expressed as a set of mutually incoherent orthogonal modes $M_i$ and the measured intensity can be described as follows:

3 $$\begin{aligned} I = \sum _{i}{\left| \mathscr {P} \left\{ O \cdot M_i \right\} \right| ^2}. \end{aligned}$$

Furthermore, the probe positions in the sample plane $r_j$ may experience slight pointing fluctuations and can thus be determined only with some experimental uncertainty. THey may need to be treated as optimizable variables during the reconstruction process. We utilized the differentiable affine transformer^2^ to calculate the transfer function of the sample at the positions of the different beamlets. In this formalism a shifted sample transfer function can be represented as follows:

4 $$\begin{aligned} O_\mathbf{r _{j}} = \mathscr {A}_{\theta _j}(O), \end{aligned}$$

where $\mathscr {A}_{\theta _j}$ represents the affine transform operator $\mathscr {A}$ driven by its parameter matrix $\theta$^2,20^. This operator is differentiable with respect to both coordinates, position $\mathbf{r} _{j}$ and object function *O*. Thus, the set of the correct probe positions $\mathbf{r} _{j}$ can be found through the gradient-based optimization of $\theta _j$.

### Single-shot ptychography

Single-shot ptychography further develops classical ptychography by recording the entire set of diffracted intensities simultaneously, utilizing a single FEL pulse rather than scanning the set of sample positions consecutively. This can be realized by splitting the probe *P* into several beamlets $B_j$, that mutually overlap on the sample surface, using a 2D diffraction grating. For successful phase retrieval in single-shot ptychography, two key prerequisites need to be met for the measured intensity distribution. First, a functional dependence has to exist between the probe and the beamlets: $B_j = g_j(P)$, which links the initial probe *P* with the j-th beamlet $B_j$. In our case, using a 2D grating and fulfilling the conditions for a paraxial approximation, all beamlets can be viewed as the original probe scaled with different intensity coefficients $\alpha _j$ corresponding to the diffraction efficiency of the grating $B_j = \alpha _j P$. The intensity distributions produced by the grating and supporting this assumption are presented in the supplementary materials (Section S1). Due to this dependency, the forward model can be expressed directly with respect to the probe *P*. Thus, there is no need for optimization of each of the beamlets $B_j$, which greatly reduces the number of optimizable variables and ensures convergence. As a second prerequisite, the diffracted intensity of each of the exit-waves of the beamlets should ideally be localized and separable from its neighbors without cross-talk. Thus, each localized intensity $I_{j} \in I$ can be attributed to a unique beamlet. When both conditions are fulfilled, the measured intensity can be numerically formulated as follows:

5 $$\begin{aligned} I_{meas} = \sum _{j}^{N}{I_j} = \sum _{j}^{N}{\left| \mathscr {P}\left\{ B_j \cdot O_\mathbf{r _{j}} \right\} \right| ^2} = \sum _{j}^{N}{\left| \mathscr {P}\left\{ \alpha _j P \cdot O_\mathbf{r _{j}} \right\} \right| ^2}. \end{aligned}$$

Both key criteria are held in our proposed scheme as follows: an FEL probe is focused by the KB mirrors downstream of the sample. The probe is split by a 2D diffraction grating placed in the vicinity of the sample. The grating-detector distance and the focal distance of the KB are set appropriately in combination to obtain an overlap of $\sim 80\%$ at the sample plane. The grating parameters are chosen adaquately to match the produced diffraction pattern to the detector chip and pixel sizes. The resolution of the reconstruction is limited by the highest scattering angle registered for a particular beamlet. When the illumination is arranged in a $x\times x$ square and a single-step propagation is utilized between the sample and detector planes, the maximum resolution of the ptychography reconstruction can be estimated as $\frac{\lambda z_{sd} dx}{Np}$, where $\lambda$ is the wavelength, $z_{sd}$ is the sample-detector distance, *Np* is the number of pixels of the detector, and *dx* is the detector pixel size^14^. However, it is possible to improve the resolution by utilizing a two-step propagation^21,22^ with the intermediate plane located at a distance $z_{si} < z_{sd}$ downstream of the sample plane. The resulting resolution of this reconstruction method can be estimated as $\frac{z_{si} dx}{z_{id}}$. In this case, the maximum achievable resolution is limited by the smallest $z_{si}$ still resulting in an adequately sampled probe wavefield in the sample plane. A detailed comparison of the reconstructions with different propagators can be found in the supplementary material (Section S2). The total number of measurable localized diffraction patterns is set by the parameters of the beam-splitting grating in combination with the detector parameters which define the trade-off between the FOV, the maximum achievable resolution, and the ptychographic oversampling requirement^23,24^.

The design of the beam-splitting grating should facilitate a clear separation of the beamlet diffraction patterns at the detector plane, which minimizes the cross-talk and leads to maximum spatial resolution in the reconstruction. Additionally, the resulting diffraction efficiency of the grating orders should be set as evenly as possible to reduce the required dynamical range of the detector. Further details on the grating parameters can be found in the “Experimental setup”. Optimization of the grating allowed us to simultaneously measure diffraction from (0, 0) up to (2, 3) grating order depending on the detector chip size. Thus, at a given wavelength, the angular size of the detector chip is the main factor limiting the number of recorded beamlets and the resulting FOV of the reconstruction.

### Single-shot forward model

Our forward model for single-shot ptychography approximates the intensity $I_j$ produced by the *j*-th beamlet $B_j$ as follows:

6 $$\begin{aligned} I_j = \alpha _j\sum _{i}{\left| \mathscr {P}_2 \left\{ \mathscr {A}_{\theta _j}(O)\cdot \mathscr {P}_1 \left\{ M_i\cdot S\right\} \right\} \right| ^2}, \end{aligned}$$

where $\mathscr {P}_1$ is the Fresnel transfer function propagator in the near field^21^ describing the propagation of the probe from the grating plane to the object plane, $\mathscr {P}_2$ is the two-step Fresnel propagator in the intermediate field^21,22^ describing the propagation of the exit-wave from the sample plane to the detector plane, *S* is the support representing the active area of the grating, $\mathscr {A}_{\theta _{j}}$ represents an affine transform and $\alpha _j$ is the scaling coefficient representing the grating efficiency for the *j*-th diffraction order. $\mathscr {P}_1$ and $\mathscr {P}_2$ are implemented as follows:

7 $$\begin{aligned}&\mathscr {P}_1(\Psi _{\rho _{g}}) = \Psi _{\rho _{s}} = \mathscr {F}^{-1}\left\{ \mathscr {F} \left\{ \Psi _{\rho _{g}}\right\} \cdot e^{(jkz_{gs})} \exp {(-j\pi \lambda z_{gs} (q_x^2+q_y^2))} \right\} \end{aligned}$$

8 $$\begin{aligned}{}&\mathscr {P}_2(\Psi _{\rho _{s}}) = \Psi _{\rho _{d}} = -\frac{i}{\lambda z_{id}}\exp (i\frac{k}{2z_{id}}\rho _d^2)\cdot \mathscr {F}\left\{ \exp (i\frac{k}{2z_{id}}\rho _i^2) \cdot \left[ -\frac{i}{\lambda z_{si}}\exp (i\frac{k}{2z_{si}}\rho _i^2)\cdot \mathscr {F}\left\{ \exp (i\frac{k}{2z_{si}}\rho _s^2) \Psi _{\rho _s}\right\} \right] \right\} , \end{aligned}$$

where $\Psi _{\rho _{g}}$,$\Psi _{\rho _{s}}$,$\Psi _{\rho _{d}}$ are complex wavefields in the grating, sample, and detector planes respectively, $z_{gs}$, $z_{si}$, and $z_{id}$ are grating to sample, sample to intermediate plane, and intermediate plane to detector distances respectively, $k = \frac{2\pi }{\lambda }$ is the wave number, $\rho = (x,y)$, $\rho _{g}$, $\rho _{s}$, $\rho _{i}$ and $\rho _{d}$ denote the transverse coordinates at the grating, sample, intermediate, and detector planes respectively, $\mathscr {F}$ and $\mathscr {F}^{-1}$ denote the forward and inverse Fourier transform while $q_x,~q_y$ are the coordinates in the Fourier space. To optimize the sampling, the intermediate plane was selected to be at the focal plane at a distance $z_{si} = 9$ cm downstream of the sample. The use of the two-step propagator allowed to have a numerical resolution of $2.1~\mu m$ and $1.9~\mu m$ for the Andor and Percival detectors, respectively. A comparison of the reconstructions with two-step and single-step propagators can be found in the supplementary materials (Section S2).

Thus, during the reconstruction the following parameters are optimized: object function *O*, probe modes $M_i$, scan coordinates $\varvec{r}_j$ expressed through the affine parameters $\theta _j$, grating-sample distance $z_{gs}$ included into the ASM propagator $\mathscr {P}_1$, and order-specific diffraction grating efficiency $\alpha _j$. The optimization was performed using the ADAM^16^ optimizer. The algorithm of the AD-powered ptychographic reconstruction is illustrated below.
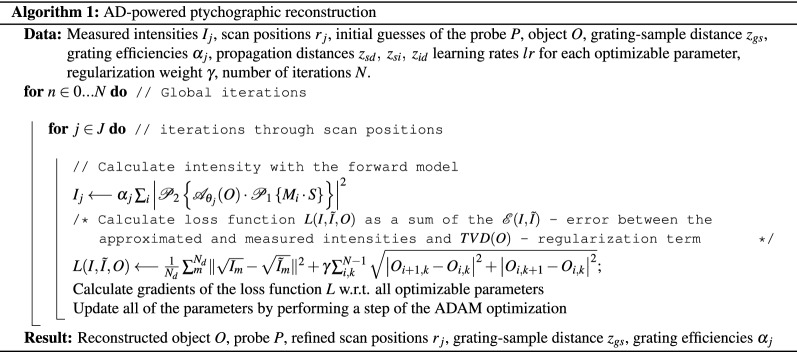


## Experiment and results

### Experimental setup

**Figure 2:**
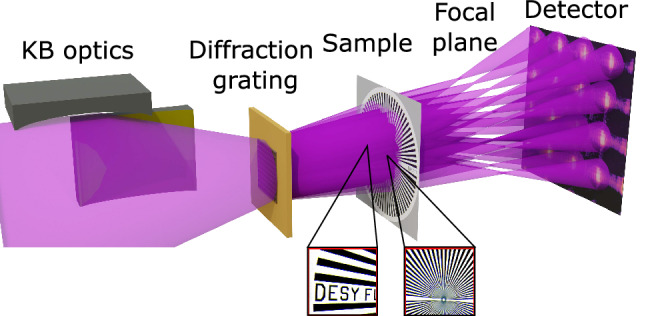
Schematic of the experimental setup. The FEL beam was focused 9 cm after the sample using bendable KB mirrors. The beam-splitting 2D diffraction grating was placed 210 cm after the center of the last KB mirror. The sample was placed approximately 0.9 cm behind the grating and thus illuminated by the overlapping beamlets produced by the grating. The two areas of the Siemens star sample ‘letters’ and ‘stripes’ are shown in the insets. The ANDOR iKon-M SO CCD was placed in the intermediate field 66 cm downstream of the sample. Alternatively, the PERCIVAL detector was placed in the intermediate field 135 cm downstream of the sample.

As a proof of principle, the proposed concept for single-shot ptychography was realized at the soft X-ray FEL FLASH. The experiment was performed at the FLASH2 beamline FL24 using a wavelength $\lambda = 13.5$ nm i.e. a photon energy $E_{ph} =91.8~eV$. The experimental setup is shown in Fig. 2.

Metal foil filters in combination with a gas attenuator^10,25^ were used to attenuate the fundamental photon beam energy to appropriate levels and to suppress higher FEL harmonics. The FEL beam was focused using a pair of bendable KB mirrors^26^ to a focal spot position of 9 cm behind the sample^10,11^. A 2D beam-splitting transmission-grating with a period $L = 2.930\,\upmu$m, an opening size $G= 0.732\,\upmu$m and an active area of $200\times 200\,{\upmu \text {m}}^2$ was placed 210 cm after the center of the last KB mirror. The grating was made from a 200 nm thick gold layer electroplated onto a 50 nm silicon nitride membrane. The active area of the grating was significantly smaller $(\sim 1/4)$ than the beam cross-section at the grating plane to select the most intense part of the beam and increase the spatial coherence. SEM images of the grating and intensity distributions measured without the sample can be found in the supplementary information (Section S1). The Siemens star sample was placed 0.9  cm downstream of the grating, which led to an average mutual overlap of $80\%$ between neighboring beamlets in the sample plane. The diffraction patterns were measured using two detectors: an ANDOR iKON-M SO CCD camera ($1024\times 1024$ pixels, $13\times 13\,\upmu \text {m}^2$ each) and the novel PERCIVAL detector ($1440\times 1484$ pixels, $27\times 27\,\upmu \text {m}^2$ each)^27^. The PERCIVAL detector offers an exceptionally high dynamical range ($5\times 10^4$ photons at $E_{ph} = 100\,\text {eV}$) and large chip size ($3.8\times 4\, \text {cm}^2$). The sample-to-detector distances were selected to fit at least $4\times 4$ beamlets onto the detector chip. This was 66 *cm* for the ANDOR camera and $135~\text {cm}$ for the PERCIVAL detector.

### Data treatment and reconstruction

**Figure 3:**
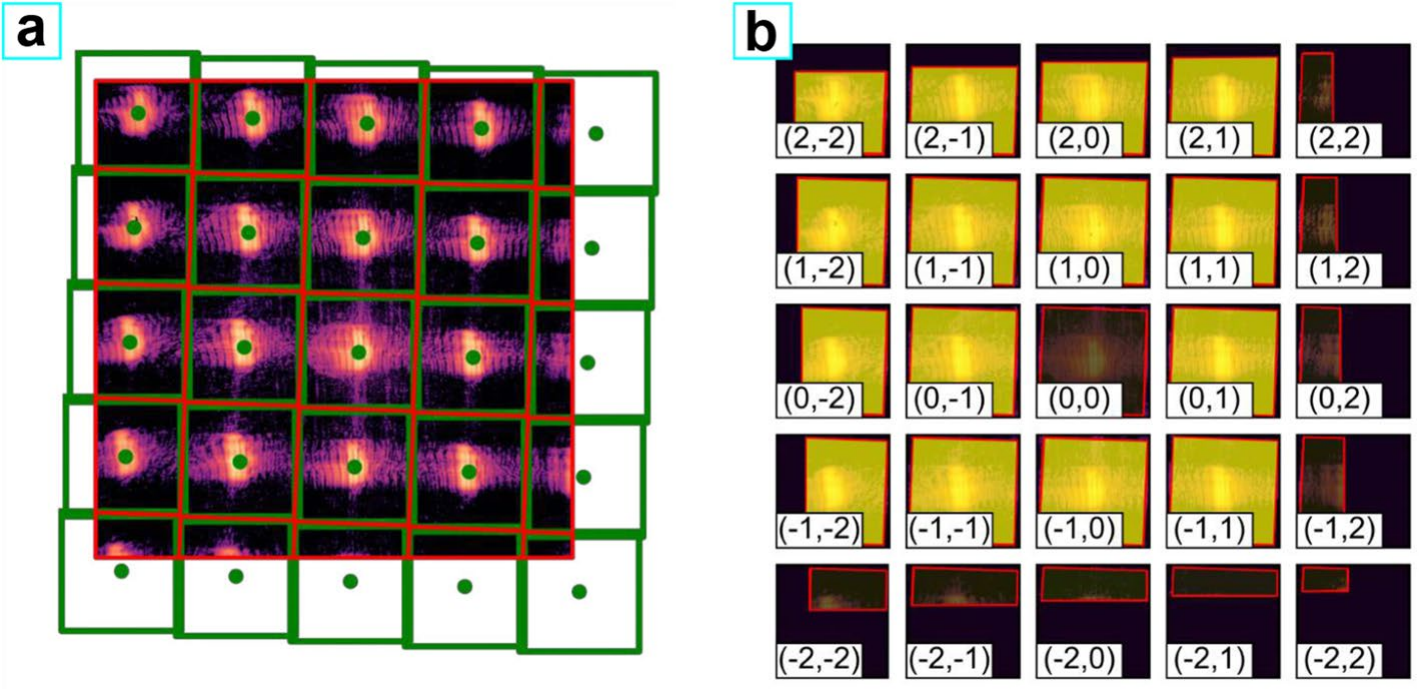
Illustration of the segmentation of the diffraction pattern. (**a**) Voronoi tessellation of the measured data. Centers of masses of sub-patterns corresponding to each of the grating orders are shown with green dots. Red polygons represent individual Voronoi cells fitting in the area of the detector chip. Green squares show the smallest square simultaneously circumscribed about and concentric with the largest Voronoi cell. (**b**) Split data used for the reconstruction. The area highlighted in yellow is inside the corresponding Voronoi cell and is constrained during the reconstruction. Each individual square is equivalent to a measurement from an individual beamlet and represents the individual computational frame used for the propagation of the respective diffraction order. The coordinates of the individual diffraction order at the sample plane can be estimated using the grating-sample distance and the grating parameters. All the data pieces with the less than half of the pixels measured were ignored during the reconstruction (right- and lower- most squares, dark in (**b**)). The (0, 0) order was also ignored during the reconstruction due to high levels of parasitic scattering from the beam passing through the frame of the grating.

In single-shot ptychography, measured intensities must be properly tessellated and then segmented into separate diffraction patterns each attributed to a single beamlet. In the next step, the patterns are placed in equal-sized computational frames used for the numerical light propagation. A possible angle between the grating and detector axes, which is experimentally difficult to avoid, prevents the use of any tessellation strategy based on the grating-detector geometry. To perform the data processing, we adopted a segmentation routine proposed by Barolak et al.^8^ which is based on Voronoi tessellation. This algorithm finds so-called Voronoi cells $v_j$ for a given set of the points $p_j$, where each cell is defined as a set of detector pixels that are closer to one particular point than to any other. The process of data segmentation is illustrated in Fig. 3.

The orientation of the grating axes with respect to the detector axes can be found by utilizing the centers of mass of the intensity patterns produced by the individual diffraction orders (shown by the green dots in Fig. 3). The horizontally and vertically diffracted orders ((0, *x*) and (*x*, 0) diffraction orders) form a basis of the grating coordinate system and thus can be used to calculate the coordinates of all the diffraction orders at the detector plane. These coordinates were used as the input points $p_j$ for the Voronoi tessellation resulting in the Voronoi cells shown with the red polygons. Each of these cells surrounds a part of the detector in which all the intensity must be attributed to a particular diffraction order. The scan coordinates at the sample plane $\varvec{r}_j$ corresponding to each order were estimated from the diffraction grating parameters and the grating-sample distance. In turn, the size of the computational frames for the light propagation was found as the smallest square simultaneously circumscribed about and concentric with the largest Voronoi cell (shown with green squares in Fig. 3). Since in some of the frames only part of the pixels (fitting inside the particular Voronoi cell shown with the red polygons in Fig. 3) could be reliably attributed to the corresponding diffraction order, the area outside the Voronoi cells (red polygons) was masked during the reconstruction.

**Figure 4:**
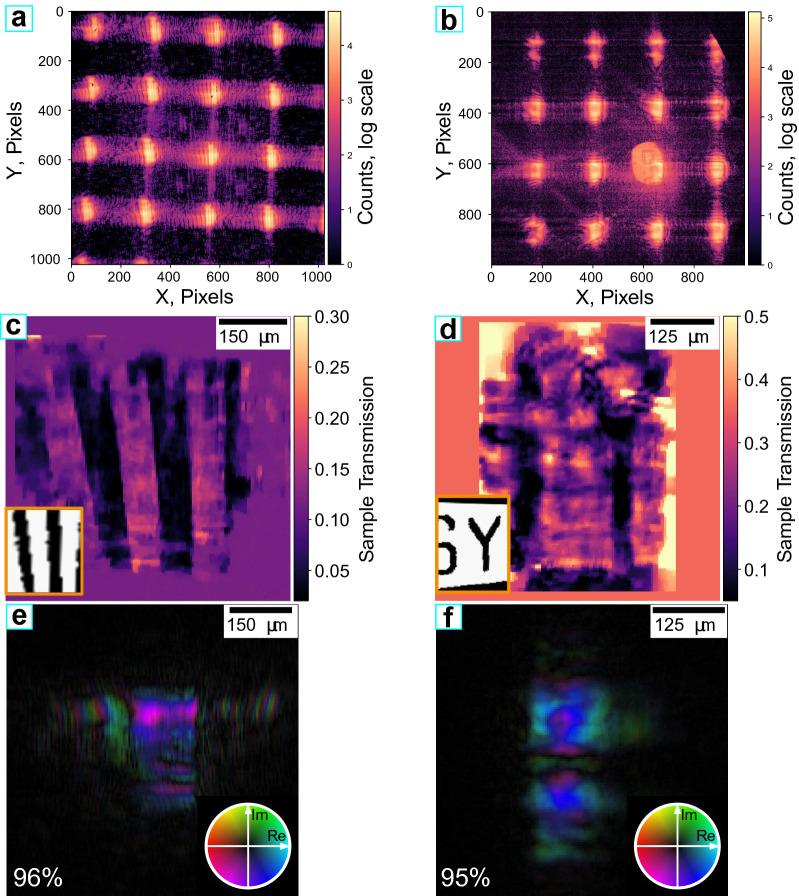
Raw data and results of the single-shot ptychography reconstructions. (**a**,**b**) Raw data used for the reconstructions of the (**c**)—‘stripes’ and **(d)**—‘letters’ regions of the sample. (**a,b**) were measured using the Andor and the PERCIVAL detector respectively. (**c,d**) Reconstructed sample transmission for (**c**)—‘stripes’ and (**d**) ‘letters’ regions of the Siemsens star sample. (**e**,**f**) Reconstructed complex wavefields of the main probe mode corresponding to (**b**,**c**), respectively. Percentages in the lower left corner represent the occupancy of the main mode.

The segmented data was used as the input for the single-shot ptychography reconstruction performed using an AD-based forward model. Diffraction patterns of the grating measured without the sample in place were used to initialize the probe modes $M_i$ and grating efficiencies $\alpha _j$. The approximate complex wavefields of all the diffraction orders at the detector plane were calculated utilizing the known defocus produced by the KB. The back-propagated wavefields were orthogonalized using a singular value decomposition and used as the initial values for the $M_i$. The diffraction efficiencies of the grating $a_j$ were estimated as the ratio of the intensity of the (0, 0) order to the intensities diffracted to the other orders. At the same time, the initial coordinates of the beamlets at the sample plane $r_j$ were estimated from the known grating parameters and grating-sample distance $z_{gs}$.

The reconstruction took $6\times 10^3$ iterations of the gradient-based optimization utilizing the ADAM optimizer. We utilized NVIDIA Tesla P100 GPU for the computations, which resulted in an iteration time of 20 ms with a total reconstruction time of 2 min. The regularization weight $\gamma$ and respective learning rates for the optimizable parameters were selected to provide fast and stable convergence.

The raw data with the averaged dark background subtracted are shown in Fig. 4. Figure 4a corresponds to the full image measured by the Andor detector, while Fig. 4b corresponds to the central part of the image measured with the PERCIVAL detector. The intensities produced by the different beamlets are separated with a limited degree of cross-talk present. The reconstructed samples and main probe modes are shown in Fig. 4. The data leading to the reconstruction of the ‘stripes’ (Fig. 4c,e) and ‘letters’ (Fig. 4d,f) areas were measured using the Andor and PERCIVAL detectors, respectively. The FLASH beam was apertured by the grating frame but nevertheless, to refrain from raising any source of uncertainty we have applied a multimodal approach. The occupancies of the main reconstructed modes are shown in Fig. 4c,d lower left corners. As expected, the results were not drastically changed using the partially coherent approximation.

**Figure 5:**
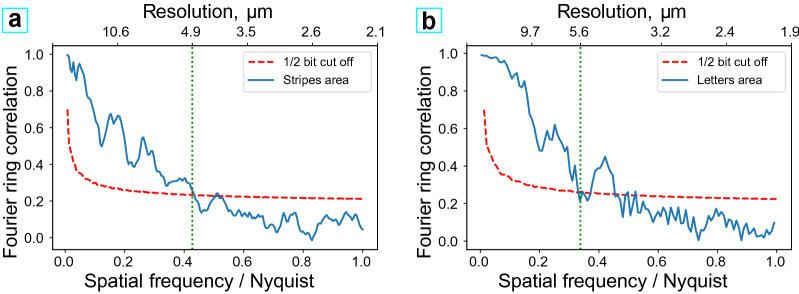
(**a**,**b**) Estimated resolutions of reconstructions using Fourier ring correlation, (**a**,**b**) correspond to Fig. 4 (**c**,**d**), respectively.

The resolution of the reconstructions was evaluated using Fourier ring correlation^28^. The resolution at the 0.5 bit cut-off was found to be approximately $4.9\,\upmu$m and $5.6\,\upmu$m for ‘stripes’ and ‘letters’ regions respectively (red dashed lines in Fig. 5a,b).

The lower resolution of the reconstruction of the ‘letters’ area can be explained by discontinuities in the probe wavefield (Fig. 4f) resulting from the shape of the SASE pulses during the measurement of the ’letters’ area.

## Discussion

Splitting of the measured diffraction pattern was performed utilizing Voronoi tessellation. This method does not require any a priori assumptions about grating to detector orientation and allows an automatic data treatment. Moreover, in the general case, this method gives the most optimal partition since any derivation from the Voronoi cells will result either in increasing the cross-talk or in reducing the maximum achievable resolution due to decreasing of the maximum registered angle of diffraction from the sample.

The reconstruction was initiated using an unknown probe wavefield and grating transfer function. The initial probe guess was obtained from the averaged intensity measured without the sample and back-propagated to the sample plane. The grating transmission function was assumed to affect only the intensity of the particular beamlet under the paraxial approximation. However, the quality of the reconstruction may be improved by performing a preliminary beam and grating characterization and taking the non-paraxial effects^29^ into account. This could be achieved by additional scanning ptychography measurements done on the beam-splitting grating itself with the same setup. However, this would require placing the grating on a high-precision motorized stage to scan the grating itself with nanometer resolution, as described in Kharitonov et al.^2^, thus increasing the complexity of the experimental setup.

Imaging in the intermediate field geometry resulted in interference between neighboring beamlets and thus a cross-talk when splitting the data for reconstruction. These effects can be avoided by placing the detector closer to the focal plane of the KB, thus resulting in a lesser degree of cross-talk. However, this would require a much higher dynamical range of the detector to measure the intensity distribution at higher scattering angles. Another possible approach could be to formulate an alternative forward model capable of simulating the interference between neighboring beams. The overall diffraction pattern can be expressed as the intensity of the coherent sum of separately propagated complex wavefields interfering at the detector plane. This formalism can integrate the inter-beamlet interference fringes into the forward model and increase the resolution of the reconstruction. However, this approach requires an optimization of the inter-beam phase difference, which might prevent the convergence of the reconstruction. Overall, further development of the experimental setup as well as the computational model for the single-shot ptychography can increase the achievable resolution.

A 2D transmission grating was utilized to perform the splitting of the XUV beam. This resulted in a limited grating efficiency and a high thermal load due to the absorption of FEL radiation in the non-transparent areas. Use of a phase grating may solve these problems, however, it may not be feasible for XUV wavelengths due to manufacturing restrictions. The very regular mesh-like beamlet pattern produced by the grating employed in our proof-of-principle experiment is highly symmetric and may cause raster-grid pathology^30^. Design of a more elaborate fan-out^31^ grating tailored for the particular setup and wavelength combination will result in a more asymmetric scan pattern^32^. Additionally, it will improve the separation and minimize the cross-talk between the neighboring beamlets in the detector plane. Using a grating with a smaller period and higher angles of diffraction may also simplify the intensity separation and improve the achievable resolution by increasing the maximally measured scattering angle of the sample. This, however, would require reformulation of a forward model in a non-paraxial single-shot formalism^29^.

## Conclusion

For the first time, single-shot ptychography was demonstrated at an X-ray free-electron laser. In a proof-of-principle experiment at FLASH2, single-shot ptychography was experimentally and computationally adapted to the XUV and X-ray wavelength range. A differentiable forward model, which allows analysis of highly fluctuating sources such as SASE type FELs^33^, was used for the AD-powered reconstruction of a Siemens star sample and the probe functions without requiring preliminary knowledge of the grating transfer function or prior photon beam characterization.

An improved experimental setup and an adaption of the computational model can extend the use of single-shot ptychography at soft X-ray FELs to sliced 3D ptychography imaging of extended samples, allowing much higher sample throughput than scanning methods. In combination with a pump-probe concept, the technique would allow fully utilizing the potential of the femtosecond-long FEL pulses for ultra-fast time-resolved imaging of dynamical processes.

## Supplementary Information

Supplementary Information.

## Data Availability

The datasets used and/or analysed during the current study available from Konstantin Kharitonov on reasonable request.
