# Supplementary material for: Single-shot ptychography at a soft X-ray free-electron laser
Source: Sci Rep. 2022 Aug 24;12:14430. doi: 10.1038/s41598-022-18605-x (PMC9402553; doi:10.1038/s41598-022-18605-x)
Supplement: Supplementary file 1 — Supplementary Information. [file 41598_2022_18605_MOESM1_ESM.pdf]

SUPPLEMENTARY INFORMATION - Single-shot ptychography at a soft X-ray free-electron laser

Konstantin Kharitonov[1,*], Masoud Mehrjoo[1], Mabel Ruiz-Lopez[1], Barbara Keitel[1], Svea Kreis[1], Seung-gi Gang[1], Rui Pan[1], Alessandro Marras[1,2], Jonathan Correa[1,2], Cornelia Wunderer[1,2], and Elke Plönjes[1]

[1]Deutsches Elektronen-Synchrotron DESY, Germany
[2]Center for Free-Electron Laser Science CFEL, Deutsches Elektronen-Synchrotron DESY, Germany
[*]konstantin.kharitonov@desy.de

S1 Grating design and performance analysis

The beam-splitting grating was designed to provide clear beamlet separation in the detector plane while simultaneously providing an as equalized order-specific diffraction efficiency as possible to minimize the dynamical rage required for the detector. The design was performed utilizing the transmission grating efficiency calculator of the CXRO database[1] and was optimized for the wavelength $\lambda = 13.5\ nm$. The resulting parameters of the grating were selected as: period $L = 2.930\ \mu m$, an opening size $G = 0.732\ \mu m$, and an active area of $200 \times 200\ \mu m^2$. The grating was made from a 200 nm thick gold layer electroplated on a 50 nm silicon nitride membrane. SEM images of the active area of the grating are shown in Fig. S1.

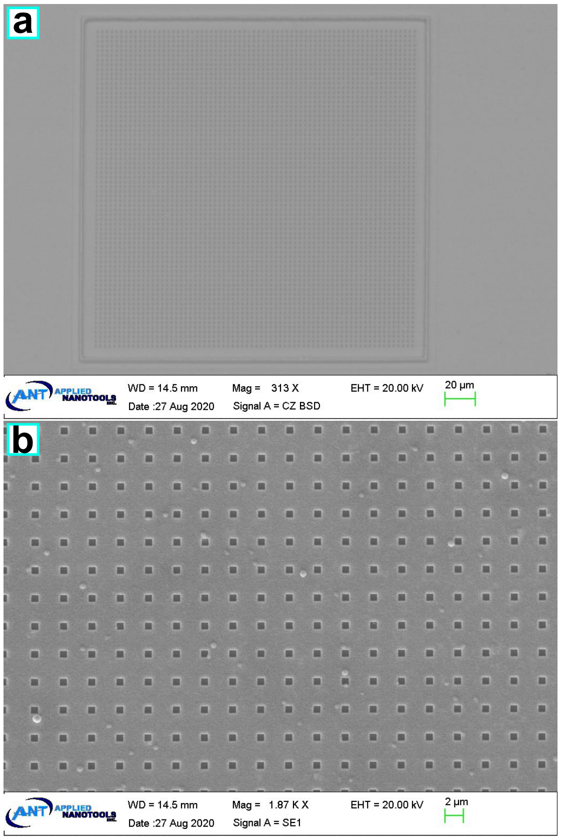

S 1. (**a**),(**b**) - SEM images of an active area of the beam-splitting grating.

The order-specific diffraction efficiency of the grating and its transfer function were studied by evaluating intensity distributions measured without the sample. They are shown in Fig. S2. A close-up view of the intensity distributions produced by the individual beamlets is shown in Fig. S3. The grating was found to have a close to ideal transfer function resulting in intensity distributions almost identical up to the multiplicative factor caused by the diffraction efficiency of the grating. Order-specific diffraction efficiencies of the grating were experimentaly measured and found to be close to the design values. They were used as the initial values in the single-shot ptychography reconstructions.

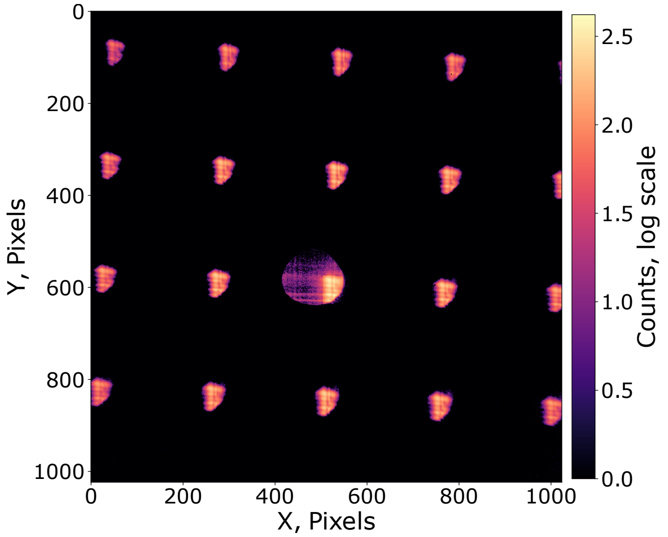

S 2. Intensity distribution measured without the sample. It was measured at a FEL fundamental wavelength of $\lambda = 13.5$ nm with fully suppressed higher harmonics. The harmonics were suppressed using metal foil filters and a gas attenuator.

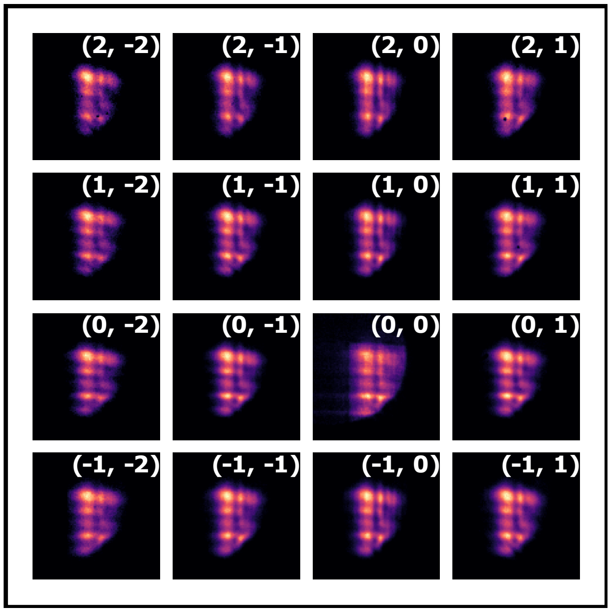

S 3. Close-up view of the intensity distributions produced by the individual beamlets. Intensity values of the individual orders are normalized to the range of $[0...1]$.

S2 Reconstructions from the simulated data

A binary 1951 USAF Target shown in Fig. S4 with transmission and phase varying from 0.2 to 0.9 and 0 to 2π, respectively, was used as the test sample. The sample was simulated on a numerical grid with a numerical resolution 669 $nm/pixel$ corresponding

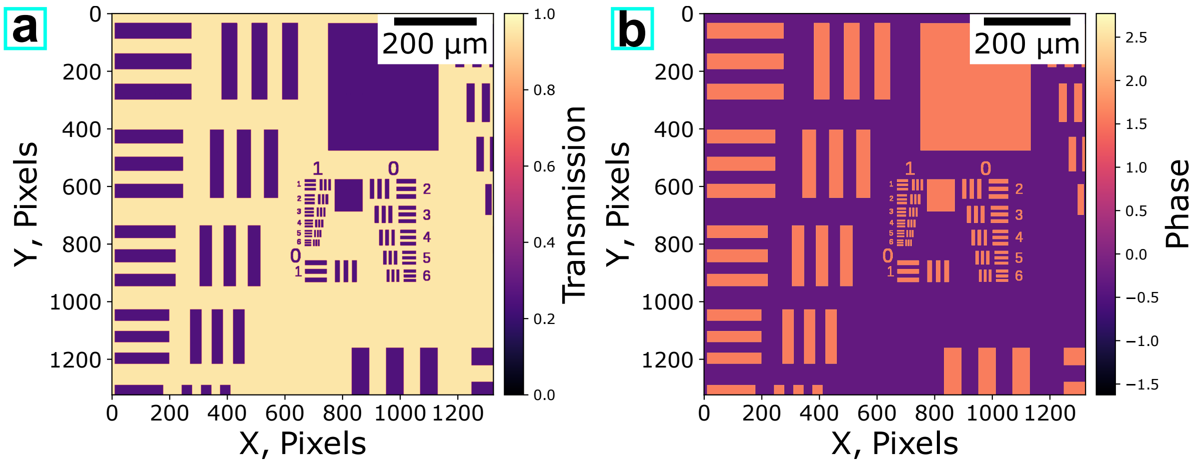

S 4. (**a**) - transmission and (**b**) phase of the simulated object

to a maximum scattering angle measured at a detector placed 66 cm downstream of the sample in the full field geometry of $dx_{samp} = \frac{\lambda z_{sd}}{N dx_{det}} = 669\ nm$. An ANDOR iKon-M ($N = 1024$, $dx_{det} = 13\ \mu m$) was used as the detector.

The probe was initialized at the grating plane with a Gaussian intensity distribution and a defocus of $16cm$. Additionally, to imitate the complex structure of the SASE beam and the effects of the KB mirror profile, the probe was modulated by a randomized speckle pattern with transmission and phase varying from 0.5 to 0.99 and $-\pi$ to π, respectively. The wavefield after the grating was calculated by aperturing the probe with a square aperture of $200 \times 200\ \mu m^2$ representing the active area of the grating. The wavefield at the sample plane was obtained by a near-field propagation through a grating to sample distance of $1\ cm$. The step size at the sample plane was estimated from the grating parameters and grating-sample distance to be $50\ \mu m$ resulting in an inter-beamlet overlap of 75% between the neighboring beamlets.

The intensity at the detector plane was calculated as the intensity of coherently summed exit-waves produced by the individual beamlets propagated to the detector as

$$I_{sum} = \sum_j \mathscr{P}_{sd} \left\{ \sqrt{\alpha_j} P O_{\mathbf{r}_j} \right\}, \tag{1}$$

where I_{sum} is the resulting intensity, $\mathscr{P}_{sd}$ is the sample-detector propagator, α_j is the diffraction efficiency of the grating for the j-th beamlet, P is the beamlet wavefield, and $O_{\mathbf{r}_j}$ is the object transmission function at position $\mathbf{r}_j$. The resulting intensity with Poisson noise applied is shown in Fig. S5.

For the reconstruction, the measured intensity distribution was separated into 16 individual intensity regions of 256×256 pixels corresponding to the individual beamlets. The separation was performed by an algorithm based on the Voronoi tessellation proposed in Barolak et al.[2].

Individual intensity regions were used as the input data for the automatic differentiation (AD) based reconstruction algorithm described in the main body of this paper. The forward model describing the intensity I_j produced by the j-th beamlet was formulated as

$$I_j = \alpha_j \left| \mathscr{P}_2 \left\{ \mathscr{A}_{\theta_j}(O) \cdot \mathscr{P}_1 \left\{ P \cdot S \right\} \right\} \right|^2, \tag{2}$$

where $\mathscr{P}_1$ is the Fresnel transfer function propagator in the near field[3] describing the propagation of the probe from the grating plane to the sample plane, $\mathscr{P}_2$ is the propagator describing the propagation of the exit-wave from the sample plane to the detector plane, S is the support representing the active area of the grating, $\mathscr{A}_{\theta_j}$ represents an affine transformation and α_j is the scaling coefficient representing the grating efficiency for the j-th diffraction order.

To illustrate the effect of different choices for the propagator $\mathscr{P}$ on the achievable resolution, we performed two reconstructions with a differently formulated propagator $\mathscr{P}$. The first 'single propagation' formulation utilizes a single Fresnel propagation as follows:

$$\mathscr{P}(\Psi_{\rho_s}) = \Psi_{\rho_d} = -\frac{i}{\lambda z_{sd}} \exp(i\frac{k}{2z_{sd}}\rho_d^2) \cdot \mathscr{F}\left\{ \exp(i\frac{k}{2z_{sd}}\rho_s^2)\Psi_{\rho_s} \right\}, \tag{3}$$

where $\Psi_{\rho_s}, \Psi_{\rho_d}$ are complex wavefields in the sample, and detector planes respectively, z_{sd} is the sample-detector distance, $k = \frac{2\pi}{\lambda}$ is the wave number, ρ_s and ρ_d denote the transverse coordinates at the sample and detector planes, respectively, and

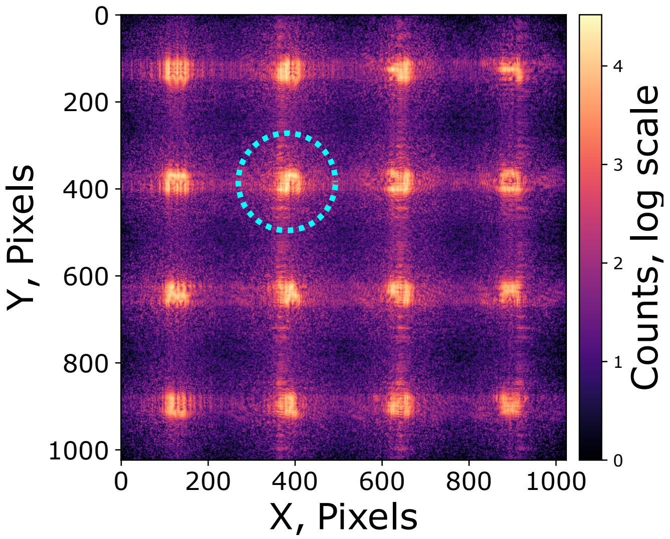

S 5. Simulated intensity distribution at the detector plane with Poisson noise applied. The (0,0) diffraction order is shown with a turquoise circle.

$\mathscr{F}$ denotes the forward Fourier transform. The numerical resolution in the sample plane achievable with this propagator can be calculated as $\frac{\lambda z_{sd}}{Ndx} = 2.67\ \mu m$, where $N = 256$ is the column/row size of the intensity region attributed to the particular beamlet and $dx = 13\ \mu m$ is the pixel size of the detector.

Alternatively, the propagator can be formulated as a 'two-step' propagation with the intermediate propagation plane ρ_i placed at the distance z_{si} downstream of the sample plane. In this case the propagation can be calculated as:

$$\mathscr{P}_2(\Psi_{\rho_s}) = \Psi_{\rho_d} = -\frac{i}{\lambda z_{id}}\exp(i\frac{k}{2z_{id}}\rho_d^2)\cdot\mathscr{F}\left\{\exp(i\frac{k}{2z_{id}}\rho_i^2)\cdot\left[-\frac{i}{\lambda z_{si}}\exp(i\frac{k}{2z_{si}}\rho_i^2)\cdot\mathscr{F}\left\{\exp(i\frac{k}{2z_{si}}\rho_s^2)\Psi_{\rho_s}\right\}\right]\right\}, \quad (4)$$

where ρ_i denote the coordinate grid in the intermediate plane placed a distance of z_{si} downstream of the sample. The 'two-step' propagator allows varying the numerical resolution in the sample plane as $\frac{z_{si}dx}{z_{id}}$, where dx is the detector pixel size. This way it is possible to get a higher resolution in comparison to the 'single-step' propagator (Equation S3). However, the propagation distance z_{si} cannot be decreased indefinitely, since the probe still needs to fully fit into the computational frame and all the phase terms need to be sampled properly to perform the propagation. In this simulated reconstruction, the intermediate distance was selected as $z_{si} = 6.16\ cm$ resulting in a numerical resolution of $1.34\ \mu m$ in the sample plane, which is two times higher than the one obtainable with the single propagation.

Samples reconstructed with the 'single-step' and the 'two-step' reconstructions are shown in Fig. S6 and S7, respectively. Both of the reconstructions lasted for 10^4 iterations ($\sim 3\ min$ of computational time). Sample O, probe P, sample translations $\mathscr{A}_{\theta_j}$, and order-specific diffraction grating efficiencies α_j were optimized during the reconstruction. It is clearly seen that both of the reconstructions converged to the image of the sample and that the use of the 'two-step' propagator allows for obtaining a higher resolution of the reconstruction. We attribute the periodic artifacts appearing in the form of periodic bright dots in Fig. S6(d) and stripes in Fig. S7(a,b) to 'raster grid pathology'[4] emerging from the ideal scan grid used in the simulations.

Additionally, total variation denoising (TVD) of the sample was performed starting from the 5×10^3rd iteration. The weight of the TVD term was initially selected to be one order of magnitude lower than the value of the loss function at the 5×10^3rd iteration. The reconstruction results obtained with the use of TVD are shown in Fig. S6 and S7 (**c**,**d**). It can be clearly seen that TVD reduces the amount of noise in the sample reconstruction while preserving the small features. The evolution of the loss function during the reconstruction is shown in Fig. S8

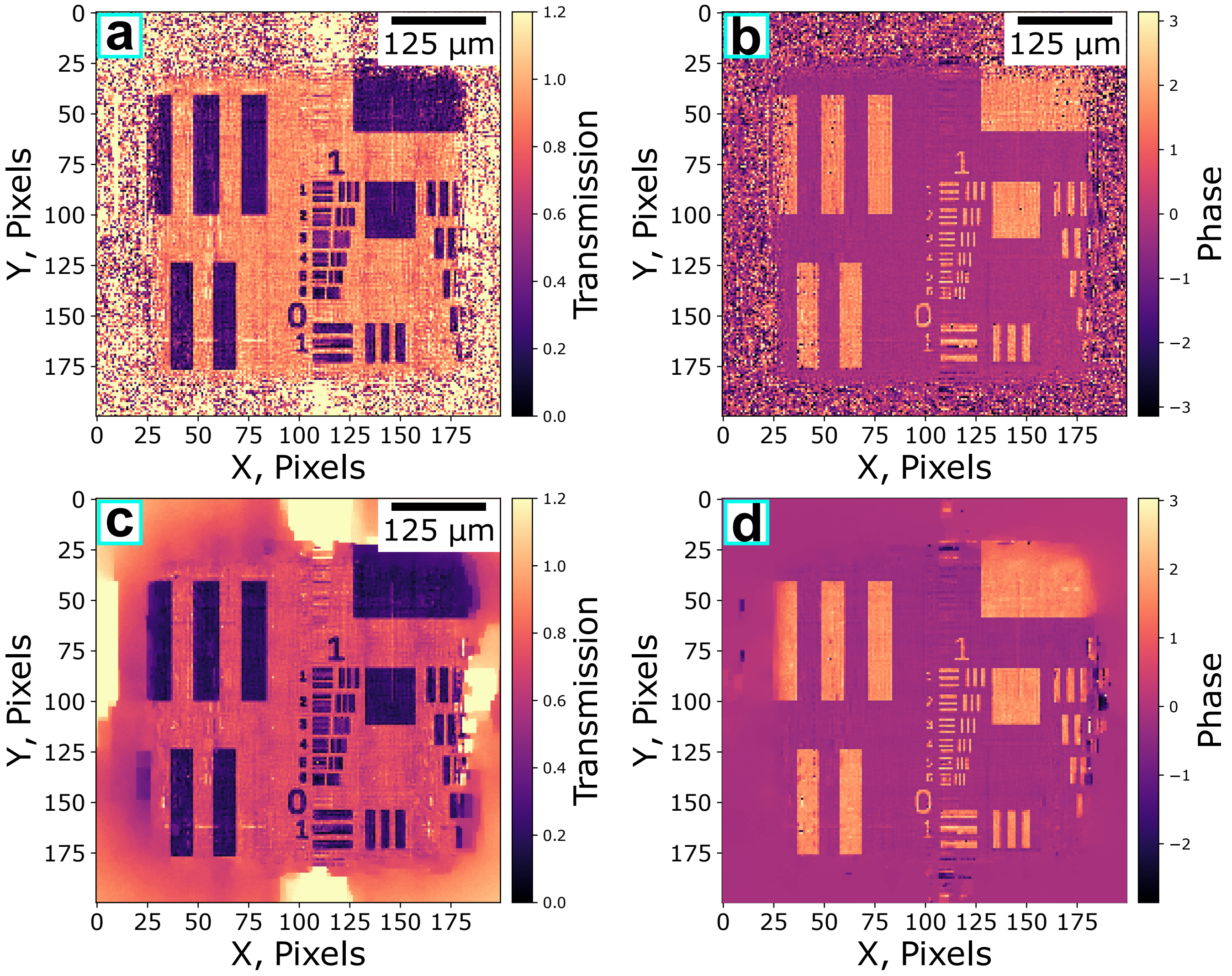

S 6. Transmission (**a**, **c**) and phase (**b**, **d**) of the sample obtained after the reconstruction utilizing the single-step propagator. (**a**, **b**) and (**c**, **d**) correspond to the results obtained without (after 5×10^3 iterations) and with the TVD regularization (after 10^4 iterations) respectively. Periodic bright spots visible in (**c**) are assumed to occur as a result of the beamlets forming a perfect square grid at the sample plane resulting in 'raster grid pathology'[4].

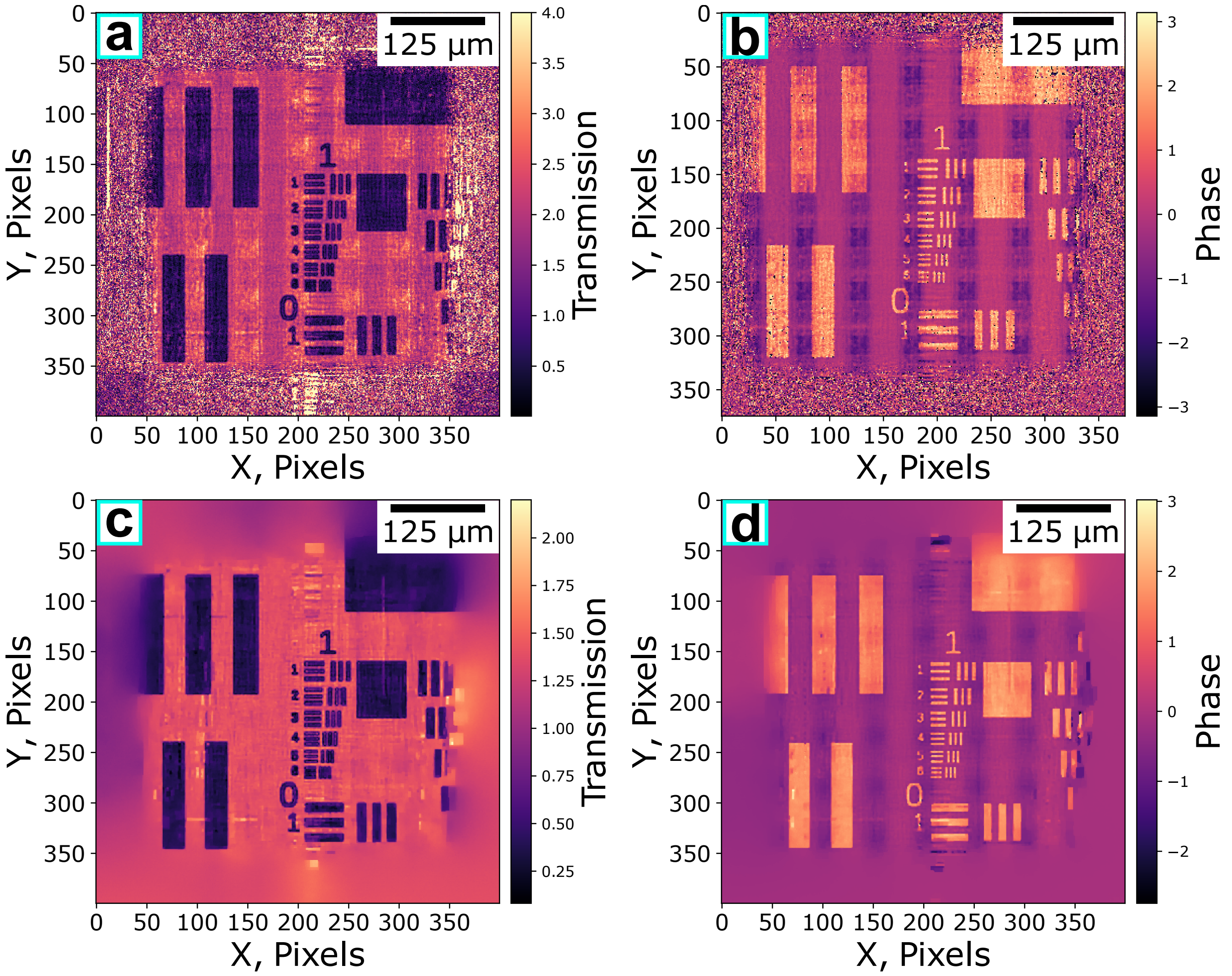

S 7. Transmission (**a**, **c**) and phase (**b**, **d**) of the sample obtained after the reconstruction utilizing the two-step propagator. (**a**, **b**) and (**c**, **d**) correspond to the results obtained without (after 5×10^3 iterations) and with the TVD regularization (after 10^4 iterations) respectively. Periodic artefacts in the reconstruction are again assumed to occur as a result 'raster grid pathology'

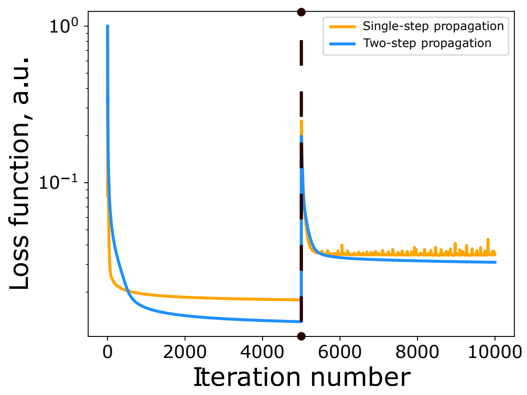

S 8. Evolution of the loss function value during the reconstruction. Orange and blue curves correspond to the reconstructions with the single-step propagator of Fig. S(6) and the two-step propagator of Fig. S(7) respectively. The reconstruction consisted of 10^4 iterations of AD-powered reconstruction algorithm. The TVD regularization was applied after 5×10^3 iterations (shown with black dashed line).)

References

1. Henke, B. L., Gullikson, E. M. & Davis, J. C. X-ray interactions: photoabsorption, scattering, transmission, and reflection at e= 50-30,000 ev, z= 1-92. *At. data nuclear data tables* **54**, 181–342 (1993).

2. Barolak, J. *et al.* Wavelength-multiplexed single-shot ptychography. *Ultramicroscopy* **233**, 113418, DOI: 10.1016/j.ultramic.2021.113418 (2022). 2009.01337.

3. Goodman, J. W. Introduction to fourier optics. 3rd. *Roberts Co. Publ.* **3** (2005).

4. Fannjiang, A. Raster grid pathology and the cure. *Multiscale Model. & Simul.* **17**, 973–995 (2019).
